# m5C methylation of mitochondrial RNA and non-coding RNA by NSUN3 is associated with variant gene expression and asexual blood-stage development in *Plasmodium falciparum*

**DOI:** 10.1186/s13071-025-06746-7

**Published:** 2025-03-27

**Authors:** Ruoyu Tang, Xuan Chen, Xiaomin Shang, Ye Hu, Binbin Lu, Xuli Du, Junlong Yang, Fengshuo Zhang, Fei Wang, Zuping Zhang, Yanli Bai, Qingfeng Zhang, Yanting Fan

**Affiliations:** 1 Department of Parasitology, School of Medicine, Xi’an International Medical Center Hospital, Northwest University, Xi’an, 710069 Shaanxi China; 2 Department of Blood Transfusion, Xi’an International Medical Center Hospital, Northwest University, Xi’an, 710069 Shaanxi China; 3 Laboratory of Molecular Parasitology, State Key Laboratory of Cardiology and Research Center for Translational Medicine, Shanghai East Hospital, Key Laboratory of Pathogen-Host Interaction (Tongji University), Ministry of Education, Clinical Center for Brain and Spinal Cord Research, School of Medicine, Tongji University, Shanghai, 200120 China; 4 Department of Parasitology, School of Basic Medical Science, Central South University, Changsha, 410013 Hunan China; 5 Traditional Chinese Medicine, Jiangxi Administration of Traditional Chinese Medicine, Key Laboratory of Pharmacodynamics and Safety Evaluation, Health Commission of Jiangxi Province, School of Basic Medicine, Nanchang Medical College, Nanchang, 330052 China

**Keywords:** *Plasmodium falciparum*, NSUN3, Variant gene, Gene regulation

## Abstract

**Background:**

Malaria is caused by *Plasmodium* spp. and is a prevalent parasitic disease worldwide. To evade detection by the immune system, by switching variant gene expression, the malaria parasite continually establishes new patterns displaying a single variant erythrocyte surface antigen. The distinct surface molecules encoded by clonally variant gene families include *var*, *rif*, *stevor*, *Pfmc-2tm*, and *surfins*. However, the mechanism behind the exclusive expression of a single member of the variant gene family is still not clear. This study aims to describe the molecular process of variant gene switching from the perspective of the epitranscriptome, specifically by characterizing the role of the *Plasmodium falciparum* RNA m5C methyltransferase NSUN3.

**Methods:**

A conditional gene knockdown approach was adopted by incorporating the glucosamine-inducible glmS ribozyme sequence into the 3′ untranslated region (UTR) of the *pfnsun3* gene. A transgenic parasite line PfNSUN3-Ty1-Ribo was generated using CRISPR-Cas9 methods. The knockdown effect in the transgenic parasite was measured by a growth curve assay and western blot analysis. The transcriptome changes influenced by PfNUSN3 knockdown were detected by RNA sequencing (RNA-seq), and the direct RNA transcripts regulated by PfNUSN3 were validated by RNA immunoprecipitation and high-throughput sequencing (RIP-seq).

**Results:**

Growth curve analysis revealed that conditional knockdown of PfNSUN3 interfered with parasite growth. The parasitemia of the PfNSUN3 knockdown line showed a significant decline at the third round of the life cycle compared with the control line. The knockdown of PfNSUN3 altered the global transcriptome. RNA-seq analysis showed that at the ring-stage depletion of PfNSUN3 silenced almost all *var* genes, as well as the guanine/cytosine (GC)-rich non-coding RNA (ncRNA) *ruf6* family. RNA RIP-seq arrays revealed that PfNSUN3 directly interacted with several *var* genes.

**Conclusions:**

Our findings demonstrate a vital role of PfNSUN3 in the process of the mutually exclusive expression of variant genes, and contribute to a better understanding of the complex mechanism of epigenetic regulation of gene expression in *P. falciparum*.

**Graphical Abstract:**

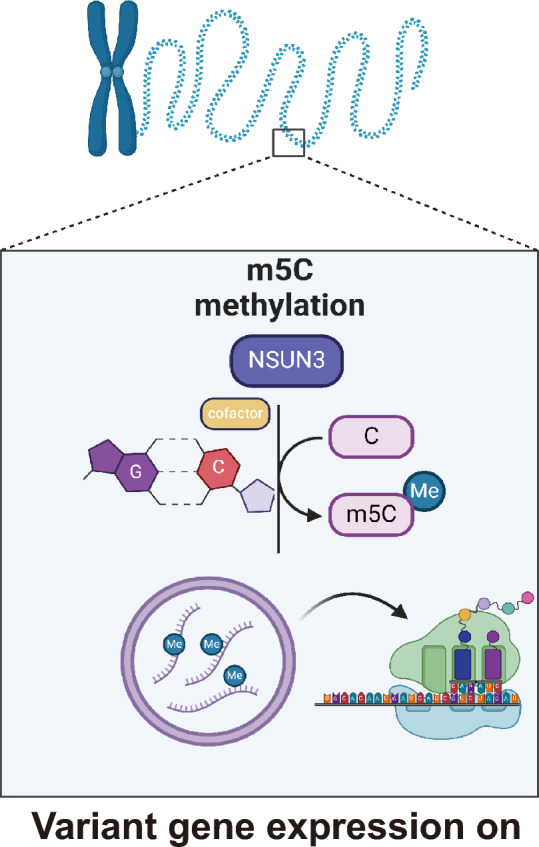

**Supplementary Information:**

The online version contains supplementary material available at 10.1186/s13071-025-06746-7.

## Background

Malaria is caused by unicellular protozoans of the *Plasmodium* genus, and is a prevalent worldwide parasitic disease. Malaria has high morbidity and mortality rates; there are approximately 247 million cases globally, with around 609,000 deaths annually [1]. After transmission via the bite of an infected mosquito and a period of development in the liver, the pathogenesis of the disease is caused by the burden of parasite invasion and development within erythrocytes. The parasite remodels the architecture of resident erythrocytes, and the human malaria parasite *Plasmodium falciparum* expresses multiple membrane proteins on the erythrocyte surface [2]. One such protein is the *P. falciparum* erythrocyte membrane protein 1 (PfEMP1), which is one of the principal antigenic substances recognized by the immune system. PfEMP1 is encoded by the *var* gene family, with ~ 60 *var* genes in the genome of the *P. falciparum* 3D7 isolate [3–5]. Through a mechanism of mutually exclusive expression, a given *P. falciparum* parasite expresses a single *var* gene and silences the remaining genes within the *var* repertoire. Thus, the malaria parasite can evade immune detection by switching *var* gene expression, and thereby continually establish new, antigenically variant PfEMP1 expression patterns [6]. *Plasmodium falciparum* also expresses other multigene virulence antigens that are exported to the surface of the red blood cell, the RIFINs, STEVORs, PfMC-2TMs, and SURFINS, which are also clonally variant [7].

Epigenetic regulation at the transcriptional level plays a critical role in the events of variant gene expression [8–10]. For example, methyltransferase PfSETvs have been studied to describe their participation in histone modifications which are involved in silencing *var* genes [11]. The architectural regulator HMGB1 is involved in virulent gene expression by establishing a high-order genome organization [12]. Post-transcriptional regulation, like the nascent RNA decay mediated by RNases, is an important process. PfRNaseII serves vital functions for *var* gene expression by directly degrading the relevant RNA. A guanine/cytosine (GC)-rich non-coding (ncRNA) (RUF6) gene family, which is located adjacent to and upstream of an active *var* gene, is directly regulated by PfRrp6 to ensure the activation of the *var* genes [13–15]. These findings demonstrate that the mutually exclusive expression of variant genes in malaria parasites is manipulated by a complex multilayered regulatory network.

Post-transcriptional modifications of RNA transcripts are important in addition to modifications at the DNA level [16–18]. Many RNA-associated methyltransferases have been identified in eukaryotes, including the NSUN family [19–22]. For example, NSUN2 in humans can promote tumorigenesis by targeting the m5C methylation site in TREX2 to restrict cytosolic double-stranded DNA (dsDNA) accumulation and cGAS/STING activation [23]. NSUN3 can drive the translation of mitochondrial RNA (mRNA) by the formation of m5C at position 34 in mitochondrial transfer RNA (tRNA)^Met^ to power metastasis [24]. Other m6A methyltransferases, such as METTL3 and the METTL3 adaptor protein WTAP, were confirmed to affect cellular and organismal processes during cell differentiation and cancer cell progression in mammalian cells [25, 26].

Recently, the methylation of adenosine (m6A) and cytosine (m5C) at internal positions within mRNA transcripts were reported as the main abundant modifications in *P. falciparum* [27]. Several methylation-related proteins were found to serve roles in the regulation of gene expression, for example, m6A methyltransferase PfMT-A70 [28] and m6A reader PfYTH.2 [29, 30]. Inverse correlations were observed between m6A methylation and mRNA stability, which were essential for parasite survival. PfNSUN2/PyNSUN2, an m5C writer, stabilizes mRNA transcripts and mediates the m5C-associated development of gametocyte production [31]. These modification forms play important roles in maintaining mRNA stability, and thereby translation. However, the function of RNA modifications in the regulation of immune evasion in malaria parasites is still unclear.

In the present study, we use RNA sequencing (RNA-seq) and RNA immunoprecipitation and high-throughput sequencing (RIP-seq) arrays to describe the function and mechanism of the putative RNA m5C methyltransferase PfNUN3 (PF3D7_1129400) in regulating variant gene expression. We found that the dysfunction of PfNUN3 influenced the variant gene expression, suggesting a key role in immune evasion during the intraerythrocytic developmental cycle (IDC). PfNSUN3 was shown to directly interact with the activated *var* gene mRNA. In addition, it was found that PfNSUN3 knockdown could affect parasite growth. Our study provides a better understanding of m5C methylation in regulating gene expression, particularly regarding the variant gene family.

## Methods

### Plasmid construction for transfection

To generate the transgenic lines PfNSUN3-HA-Ty1 and PfNSUN3-Ty1-Ribo, we modified the circular pL6CS plasmid by inserting a 1-kb-long homolog sequence flanking the C terminus of the *Pfnsun3* gene (PF3D7_1129400) and a guide RNA sequence (5′-ataaacataaatgactaaacagg-3′) specific to the *Pfnsun3* gene using the In-Fusion polymerase chain reaction (PCR) cloning system. The guide RNA was cloned into the *pL6CS* plasmid between the PstI and XhoI restriction enzyme sites. The homologous sequence of the *Pfnsun3* gene was inserted into the AscI and AflII restriction enzyme sites. Primers used for PCR amplification were as follows: LHR-F: 5′-caaaagatcagacgcagtatttac-3′, LHR-R: 5′-tactttattcttaatatttttcatattttttttattttt-3′, RHR-F: 5′-aaagtgacaatgaaatgaaattatatat-3′, and RHR-F: 5′-gcatatattcaaaacgattgagatac-3′.

### Parasite culture and transfection

The *P. falciparum* isolate 3D7-G7 was maintained in in vitro culture and synchronized according to standard procedures. Synchronized ring-stage parasites at 5% parasitemia were transfected with 100 μg of pUF-Cas9-BSD plasmid and pL6CS-pfnsun3-ty1-glms (pL6CS-pfnsun3-ha-ty1) via Bio-Rad electroporation. The transgenic cultures were selected by blasticidin S deaminase (BSD) and WR99210. After approximately 4 weeks, the positive cultures were subcloned by limiting dilution, and the integration events were verified by specific PCR amplification followed by sequencing.

### Growth curve analysis

Tightly synchronized ring-stage *Pfnsun3-ty1-glms* cultures were diluted to 0.1% parasitemia and divided into two groups in a six-well plate, and incubated with and without 2.5 mM glucosamine (GlcN). The cultures were continuously maintained for four replication cycles, and at each cycle the parasitemias were determined using light microscopy of Giemsa-stained thin blood smears. The experiment was performed in triplicate, and the data were processed using GraphPad Prism software.

### Western blot

Synchronized parasites at 5% parasitemia were collected and lysed with 0.15% saponin. Following washing with phosphate-buffered saline (PBS), the parasites were resuspended in 1× sodium dodecyl sulfate (SDS) loading buffer (Bio-Rad) and the proteins were electrophoretically separated on 10% SDS–polyacrylamide gel electrophoresis (PAGE) gels and transferred to membranes for western blot analysis. Mouse anti-Ty1 antibodies (Sigma-Aldrich) were used to visualize the approximately 73 kDa PfNSUN3 protein, and rabbit anti-PfAldolase (Abcam) was used to recognize parasite aldolase as a loading control. An ECL Prime Chemiluminescent Western Blotting kit (GE Healthcare) was used to detect the positive protein bands.

### RNA-seq and data analysis

Tightly synchronized *Pfnsun3-ty1-glmS* cultures were divided into two groups and treated with and without 2.5 mM GlcN. The total RNA samples were collected at the ring (10–15 h post-inoculation [hpi]), trophozoite (25–30 hpi), and schizont (40–45 hpi) stages of the second IDC. A Zymo RNA kit was used to extract the total RNA according to the instruction manual. Library preparation for strand-specific RNA-seq was prepared using a KAPA Stranded mRNA-Seq Kit. Libraries were sequenced on an Illumina NovaSeq 6000 system to generate 150-base-pair (bp) paired-end reads. The RNA-seq raw data were trimmed by trim-galore and aligned to the PlasmoDB-45_Pfalciparum3D7 genome using Hisat2. Read counts were calculated using FeatureCounts. Fragments per kilobase of transcript per million mapped reads (FPKM) were calculated in RStudio. Subsequently, the differentially expressed genes (DEGs) were quantified with the criteria of FPKM fold change greater than 3 or lower than −3. Gene ontology (GO) enrichment analysis was performed in PlasmoDB (https://plasmodb.org/plasmo).

### RIP-seq and data analysis

RIP assays were performed as described previously [14]. Briefly, 5 × 10^9^ synchronized ring-stage parasites were collected and treated with saponin. The parasite pellets were lysed under a non-denaturing condition. Supernatants were collected and incubated with anti-ty1 antibody and protein A/G magnetic beads. RNA was purified using TRIzol reagent and a phenol–chloroform method. The eluted RNA was directly used to prepare strand-specific RNA-seq libraries without poly (A) enrichment. Libraries were sequenced on an Illumina NovaSeq 6000 system using 150-bp paired-end reads. The RIP-seq raw data were filtered as described [13] and aligned to the genome by Hisat2. Samtools was used to convert file formats and remove duplicated reads. Sorted files were transformed into “bigwig” files by bamCoverage. Enrichment heatmaps were generated by deepTools computeMatrix and plotHeatmap tools.

## Results

### Characterization of the NSUN3 ortholog in *P. falciparum*

**Fig. 1:**
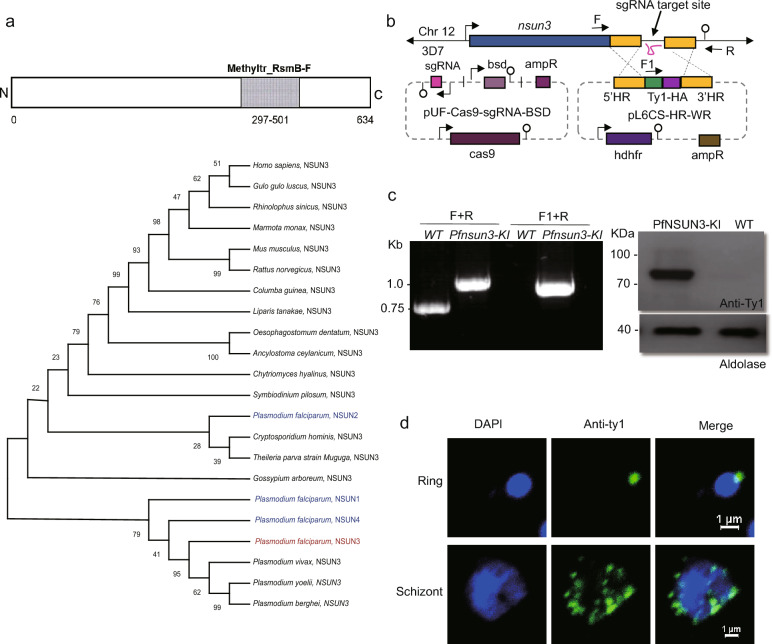
Characterization of the NSUN3 ortholog in *P. falciparum*. **a** Upper: The domain structure of the PfNSUN3 protein; the approximate size of the Methyltr_RsmB-FN domain is 204 amino acids. Lower: Phylogenetic trees of NSUN protein family orthologs in eukaryotes. **b** Schematic representation of the construction of the transgenic line PfNSUN3-HA-Ty1. Co-transfection of plasmids *pUF1-BSD-cas9* and *pL6CS-hDHFR-pfnsun3-HA-Ty1* leads to gene integration. **c** PCR and western blot analysis of the PfNSUN3-HA-Ty1 line. **d** IFA of PfNSUN3 with the PfNSUN3-HA-Ty1 line. DAPI, 4′,6-diamidino-2-phenylindole

*Pfnsun3* (PF3D7_1129400, PlasmoDB) encodes a 634-amino-acid protein, with a predicted “Methyltr_RsmB-F” catalytic domain characteristic of ribosomal RNA cysteine methyltransferases, located between amino acid residues 297 and 501 (https://www.uniprot.org). To trace the evolutionary relationship of NSUN3 proteins in eukaryotes (Table S1), including malaria parasite species, we constructed a phylogenetic tree using the whole sequences of NSUN protein ortholog. NSUN3 proteins formed a clade distinct from the NSUN family proteins NSUN1, NSUN2, and NSUN4 (Fig. 1a). A sequence alignment showed that the catalytic Methyltr_RsmB-FN domain of NSUN3 proteins is highly conserved in the *Plasmodium* genus (Fig. S1). To determine the subcellular localization of PfNSUN3, we constructed a PfNSUN3-HA-Ty1 knock-in strain. Fragments of roughly 1-kb bases before and after the stop codon of the *pfnsun3* gene were selected as homologous arm sequences, and three tandem HA and Ty1 tags were cloned into the plasmid. After transfection of the constructed plasmids into wild-type (WT) parasites, they were cultured for about 3 weeks with WR99210 and BSD drug selection until live parasites were seen by microscopy (Fig. 1b). To confirm that the parasites were successfully transformed, we designed a forward primer F upstream of the 5′ homologous arm, a forward primer F1 within the tag sequence, and a reverse primer R downstream of the 3′ homologous arm sequence. The lengths of the PCR products from WT and transgenic strains were verified using the F+R and F1+R primers and showed the successful generation of the PfNSUN3-HA-Ty1 parasite strain. Western blot assays demonstrated that PfNSUN3 had been successfully tagged by Ty1 and hemagglutinin (HA) (Fig. 1c). Immunofluorescence assay (IFA) analyses of the parasites with anti-Ty1 antibody showed that PfNSUN3 protein was present within the parasite cytoplasm throughout the IDC (Fig. 1d), which is consistent with its localization in studies of other organisms.

### PfNSUN3 dysregulation alters the global transcriptome

**Fig. 2:**
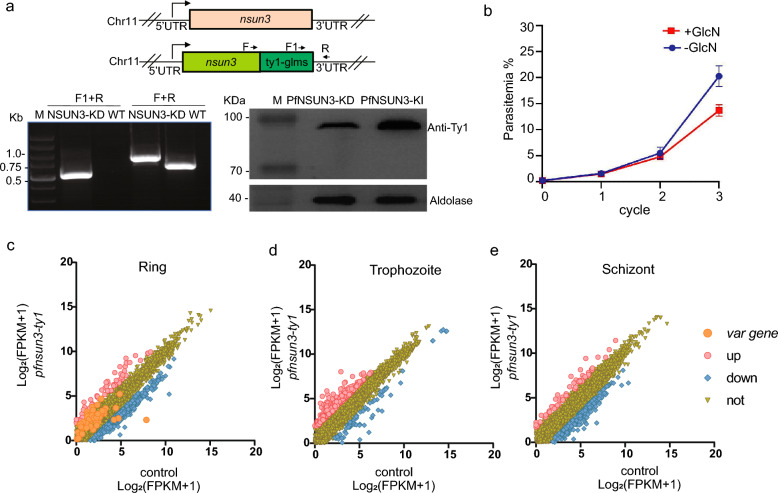
PfNSUN3 knockdown triggered alteration of the transcriptome of blood-stage parasites. **a** Upper: Schematic representation of the construction of the transgenic line PfNSUN3-Ty1-Ribo. Lower: PCR analysis of PfNSUN3-Ty1-Ribo lines (left). Western blot of PfNSUN3 protein with antibody against Ty1 for the PfNSUN3-Ty1-Ribo and PfNSUN3-HA-Ty1 lines. Aldolase was used as an internal control (right). **b** Growth curve assay of the PfNSUN3-Ty1-Ribo strain with or without culture GlcN (*n* = 3, bars indicate standard deviation [SD]). **c**–**e** Comparative transcriptome analysis of PfNSUN3-Ty1-Ribo versus WT clones at the ring, trophozoite, and schizont stages

To investigate the function of PfNSUN3 in malaria parasites, we first attempted to knock out the *Pfnsun3* gene by a frame-shift strategy using clustered regularly interspaced short palindromic repeats (CRISPR)/CRISPR-associated protein 9 (CRISPR-Cas9) methods, but failed to obtain positive lines after three independent transfection experiments. This outcome suggests that the *Pfnsun3* gene has an essential role in parasite survival. To acquire a conditional knockdown line of PfNSUN3, the GlcN-inducible glmS ribozyme sequence was incorporated into the 3′ untranslated region (UTR) of the gene. The mRNA abundance from the modified *Pfnsun3* gene would be expected to decline at the post-transcriptional level upon the addition of GlcN in the culture medium. PfNSUN3-Ty1-Ribo lines were obtained through homologous recombination caused by a double-crossover event after transfecting the recombinant plasmid *pL6cs-Pfnsun3-ty1-glms* and *pUF-Cas9* into the 3D7 strain. The selection drugs BSD and WR99210 were added to the transfection cultures until positive parasites appeared, and then limiting dilution cloning was conducted. After 22 days, we successfully obtained PfNSUN3-Ty1-Ribo transgenic parasite clones. The integration events were firstly identified by PCR (Fig. 2a).

To evaluate the knockdown effect, 2.5 mM GlcN was added to cultures of the transgenic parasite line. Western blotting analysis showed that the expression level of the PfNUSN3 protein was slightly decreased in the ring- and trophozoite-stage parasites when GlcN was present in the culture for two life cycles. However, there was no obvious difference in PfNUSN3 protein levels in schizont-stage parasites (Fig. S2a). Growth curve assays of the PfNSUN3-Ty1-Ribo lines were carried out through the IDC, and showed that the parasitemia of PfNSUN3-Ty1-Ribo lines decreased by 24% at the third cycle (Fig. 2b).

To explore the influence of PfNSUN3 knockdown on gene expression, we performed comparative transcriptome analyses of the PfNSUN3-Ty1-Ribo clone with and without GlcN treatment. Percoll-sorbitol treatment was used to obtain strictly synchronized parasites, followed by two growth cycles. The synchronized parasites were divided into two groups, with and without GlcN treatment. Total RNA was harvested for RNA-seq at the ring, trophozoite, and schizont stages. WT 3D7-G7 clones were used as controls for normalization. The comparative transcriptome analysis showed that there were no obvious transcriptomic differences upon GlcN treatment at the different IDC stages (Fig. S2b–d; Table S2). Scatter plots showed that the global transcriptome of PfNSUN3-Ty1-Ribo without GlcN was influenced compared to the WT parent strain 3D7-G7 clone (Fig. 2c–e). In detail, a total of 588, 74, and 239 genes were downregulated twofold or more at the ring-, trophozoite-, and schizont-stage parasites, respectively, while a total of 65, 305, and 60 genes were upregulated, respectively (Fig. S2f, Table S3). Western blot analysis demonstrated that the PfNSUN3 expression level was interfered with by incorporating the *glms* ribozyme sequence into the 3′ UTR (Fig. 2a). These results imply that the PfNSUN3-Ty1-Ribo line without GlcN was a PfNSUN3 mutant line. The altered expression level of PfNSUN3 influences the gene transcriptome of malaria parasites during the asexual stage.

### PfNSUN3 knockdown influences the variant gene expression

**Fig. 3:**
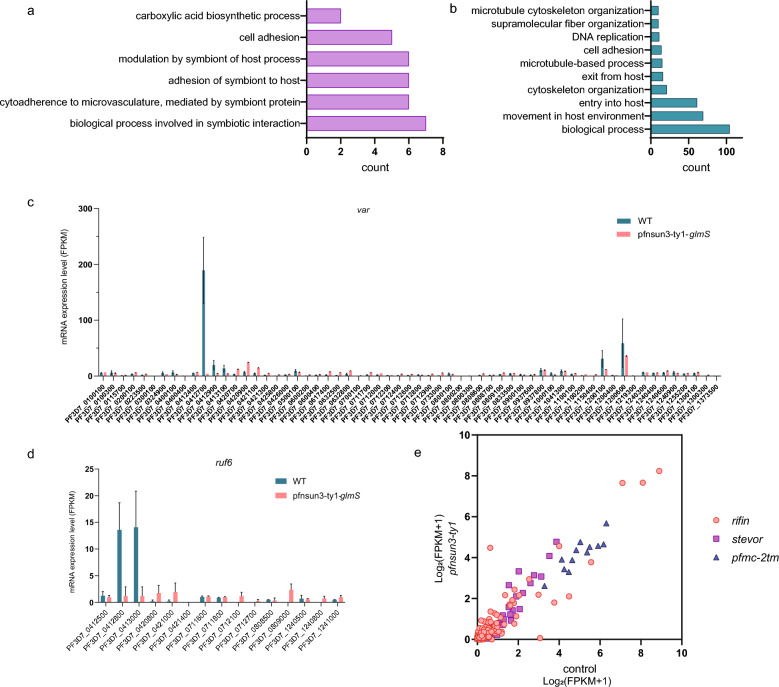
The transcriptome changes in the *var* gene family by PfNSUN3 knockdown. **a** Enriched Gene Ontology (biological processes) terms for upregulated genes by PfNSUN3 knockdown at the ring stage. **b** Enriched Gene Ontology (biological processes) terms for downregulated genes by PfNSUN3 knockdown at the ring stage. **c** The transcriptome changes in the *var* gene family of PfNSUN3-Ty1-Ribo versus WT clone at the ring stage. **d** The transcriptome changes in the *ruf6* gene family of PfNSUN3-Ty1-Ribo versus WT clone at the ring stage. **e** The transcriptome changes in the variant gene family of PfNSUN3-Ty1-Ribo versus WT clone at the trophozoite stage

Due to the significant impact of PfNSUN3 dysregulation on *P. falciparum* ring-stage parasites, we conducted an in-depth analysis of the transcriptome data. GO term analysis showed that the upregulated genes at the ring stage mostly included biological processes involved in symbiotic interaction, cell adhesion, and carboxylic acid biosynthesis (Fig. 3a). The downregulated ring-stage genes were involved in movement in the host environment, cytoskeleton organization, microtubule-based processes, cell adhesion, and DNA replication (Fig. 3b).

The activated *var* gene (PF3D7_0412700) in the parent 3D7 strain was silenced upon incorporation of the glmS ribozyme sequence (Fig. 3c); likewise, there were no transcription changes in any *var* genes with GlcN treatment versus without (Fig. S2d). The distribution of *ruf6* NC-RNA loci and the variant gene family on the chromosomes are characteristic, and we further analyzed the transcriptome level of *ruf6* genes in the PfNSUN3-Ty1-Ribo line compared to the 3D7 line. The activated GC-rich *ruf6* ncRNAs (PF3D7_0412800, PF3D7_0413000), which are dispersed within the activated *var* gene clusters, were essentially silenced after PfNSUN3 dysregulation (Fig. 3d). The transcription of *rif*, *stevor*, and *Pfmc-2tm* genes were also affected (Fig. 3e). These results indicate that PfNSUN3 is involved in the regulation of the exclusive expression of variant genes, which is crucial for maintaining the transcriptional activation of variant genes in *P. falciparum.*

### Identification of the direct substrates of PfNSUN3

**Fig. 4:**
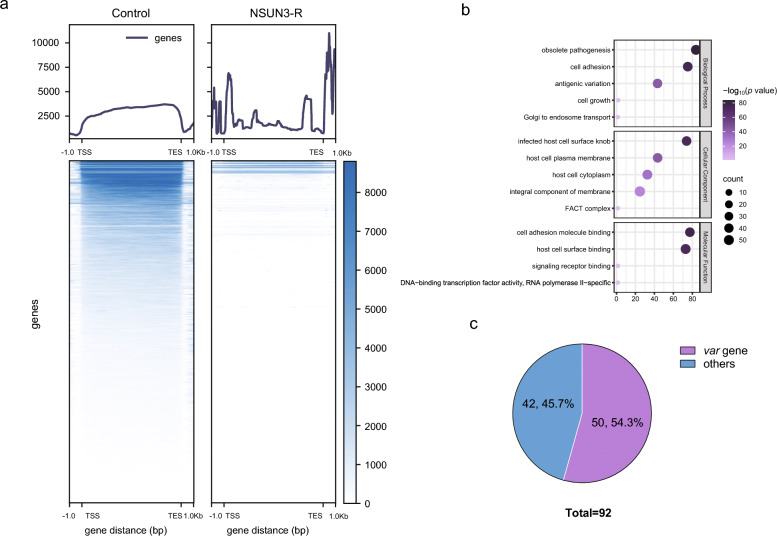
Target genes identified by PfNSUN3 enrichment. **a** Average profile of RIP/control enrichment and gene coding sequences at the ring stage. **b** Enriched Gene Ontology (biological processes) terms for the target genes obtained from RIP-seq. **c** Venn diagram showing the proportion of *var* genes among the substrates bound by PfNUSN3

The data described above demonstrate that PfNSUN3 is a vital activator factor of variant genes, but it is unknown whether PfNSUN3 catalyzes them directly. To identify PfNSUN3 substrates, we used PfNSUN3-HA-Ty1 epitope-tag transgenic lines to carry out an RIP-seq assay. Synchronized ring-stage parasites (10–15 hpi) were collected and treated with saponin, and the parasite pellets after centrifugation were lysed under a non-denaturing condition. The supernatant was collected and incubated with anti-Ty1 antibody and protein A/G magnetic beads. The eluted RNA was used to prepare strand-specific RIP-seq libraries. RIP-seq analysis showed that the large majority of binding sites of PfNSUN3 were detected within the coding sequences of the combined transcripts, mostly close to the 5′ or 3′ UTRs of the binding mRNA. Results of RIP-seq analysis identified 92 mRNA substrates after matching to the genome (Fig. 4a, Table S4). This result suggests that PfNSUN3 may modify the neighboring 5′ or 3′ UTR regions of genes, thus affecting the processes of mRNA stabilization, maturation, and translation. To clarify the type of RNA transcripts directly bound by PfNSUN3, we conducted GO enrichment analysis. Among the enriched genes captured by PfNSUN3, most belonged to host cell surface binding, antigenic variation, obsolete pathogenesis, integral component of membrane, FACT complex, signaling receptor binding, and cell growth (Fig. 4b), indicating that PfNSUN3 plays a crucial role in the interaction between the pathogen and the host. In an analysis of the RNA substrates bound by PfNSUN3, we found that the proportion of *var* genes was as high as 54%, which demonstrates that PfNSUN3 regulates transcription and translation by directly binding to the variant gene mRNA (Fig. 4c). In addition, there were five GC-rich *ruf6* ncRNAs among the enriched genes (Table S4). This phenomenon further confirms a relationship between the transcription of *var* genes and the ncRNA *ruf6* [14]. We observed that PFNSUN3 binds to the repressor *pfAP2-G2*, which is associated with *Plasmodium* sexual development [32], implying that *pfAP2-G2* also undergoes post-transcriptional regulation.

**Fig. 5:**
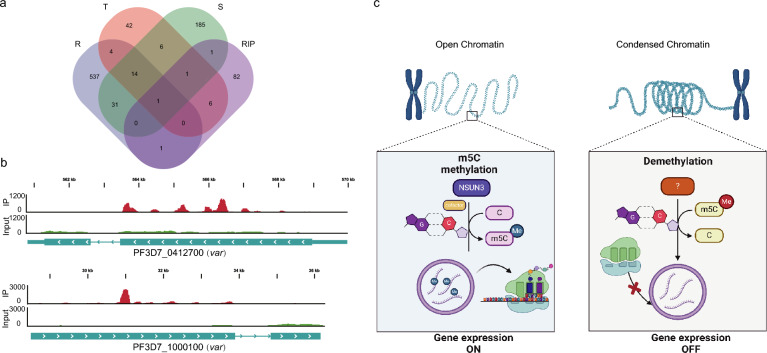
PfNSUN3 protein modifies the *var* genes directly. **a** Venn diagrams showing the intersections between target genes from RIP-seq data and downregulated genes at the R (ring), T (trophozoite), and S (schizont) stages. **b** Track view showing the enrichment signals of PfNSUN3 protein on two downregulated *var* genes. **c** Putative model of PfNSUN3 in regulating the expression of genes in the intraerythrocytic stage. In the wild-type parasites (left), PfNSUN3 modifies the mRNA, which secures the normal expression of parasite genes. When PfNSUN3 was downregulated (right), the methylation level of mRNAs was changed, thus leading to the abnormal translation of parasite mRNAs

The above RIP experiment reveals the types of RNA substrates directly regulated by PfNUN3, which provides effective data for further clarifying the target genes under its regulation. To further confirm the regulatory role of PfNSUN3 in gene expression of the malaria parasite, integrative analyses of RIP-seq and RNA-seq were conducted. A total of 10 genes were found among the intersection genes (Fig. 5a). The track view of two *var* genes, which were downregulated during NSUN3 knockdown, showed that the binding sites of PfNSUN3 mainly appeared within the 3′ region of exon 1 (Fig. 5b). These results indicated that PfNSUN3 may be involved in the maturation of mRNA, such as the removal of introns. However, this conjecture needs to be verified by further experiments.

## Discussion

The process of RNA transcriptional modification mainly involves three types of effector proteins: writer, reader, and eraser. The identified mRNA m5C writers include NSUN2, NSUN6, TRDMT1, TRM4B, and OsNSUN2; the readers include ALYREF, Y box binding protein 1 (YBX1), and RAD52; and the erasers include TET1 [33, 34]. Numerous lines of evidence elucidate a crucial role for the NSUN family in the regulation of gene expression, particularly regarding human tumorigenesis and progression. Through sequence alignment, four homologous proteins of human NSUN2 were identified in the PlasmoDB database, namely, PF3D7_0704200, PF3D7_1111000, PF3D7_1129400, and PF3D7_1230600 (PfNSUN1 to PfNSUN4). In *Plasmodium*, NSUN2 has been implicated in gametocyte production [31]. Thus, the NSUN family likely plays an indispensable role in the growth and transmission of the malaria parasite, which contributes to a better understanding of the complicated epigenetic regulation of gene expression in *P. falciparum*.

This study illustrates the function of PfNSUN3 in the regulation of gene expression. The inability to disrupt the *pfnsun3* gene in a transgenic knockout line highlights the importance of *pfnsun3* in parasite survival. *Pfnsun3* knockdown lines were obtained using CRISPR-Cas9 methods, to insert a glmS ribozyme sequence into the 3′ UTR of the gene. The knockdown efficiency of *Pfnsun3* itself did not reach a desirable level, as measured by RNA-seq. Nonetheless, the global transcriptome of the malaria parasites was significantly altered, supporting a crucial role of *Pfnsun3* in the IDC. Looking forward, it is necessary to find methods to attain efficient knockdown lines.

In this study, the majority of the identified DEGs were downregulated following PfNSUN3 knockdown, indicating a key role of PfNSUN3 in maintaining mRNA stability, which needs to be clarified by designing additional experiments. The only activated *var* gene identified in the parental 3D7 strain was completely silenced after PfNSUN3 knockdown, and PfNSUN3 was found to directly bind to *var* gene transcripts. Thus, these results show that m5C methylation plays a vital role in the immune evasion and virulence of malaria parasites, which can be better illuminated in further studies. At the same time, the transcriptional level of RUF6 adjacent to the activated *var* gene is also significantly downregulated. The results of the RIP experiment show that PfNUN3 binds directly to RUF6. Based on the above results, the decrease in the transcriptional level of RUF6 is caused by the reduction of RNA m5C methylation level due to NSUN3 knockdown rather than the spread of chromatin state. Apart from the variant genes, some essential genes of the malaria parasite were bound by PfNSUN3 and were significantly downregulated, including *g27/25*, *ap2-g*, *ap2-o*, *ap2-exp2.* These results imply that PfNSUN3 may participate in multiple cellular processes during the parasite intraerythrocytic life cycle. Human NSUN3 drives the translation of mitochondrial mRNA by the formation of m5C at position 34 in mitochondrial tRNA^Met^ [24]. In *Plasmodium*, the transcriptional levels of several mRNA were also affected after the PfNSUN3 knockdown, especially the cytochrome c oxidase subunits (Table S1). However, mitochondrial RNA were not present among the targets bound by PfNSUN3, which suggests that there may be other proteins participating in the methylation process. In summary, our data revealed the function of PfNSUN3 in *P. falciparum*, and that an adequate level of PfNSUN3 maintains the m5C methylation modification of variant genes to facilitate protein translation (Fig. 5b). These results provide a new perspective on the transcriptional regulation of variant gene expression.

## Conclusions

Here, we describe an essential function of PfNSUN3 during the IDC in the human malaria parasite, *P. falciparum*. To characterize PFNSUN3 function, we obtained an inducible knockdown transgenic line of *Pfnsun3* using CRISPR-Cas9 methods. Growth curve analysis revealed that conditional knockdown of PfNSUN3 could interfere with the growth of parasites. PfNSUN3 protein knockdown altered the global transcriptome, and at the ring stage silenced the activated *var* gene and GC-rich ncRNA *ruf6* in the parent 3D7 strain. RIP-seq arrays revealed that PfNSUN3 directly interacted with the activated *var* gene. Taken together, our findings suggest that PfNSUN3 regulates gene expression by modifying RNA transcripts and affecting RNA translation, which contributes to understanding the complex epigenetic regulation of gene expression of malaria parasites.

## Supplementary Information

Supplementary Material 1: Table S1. Protein IDs of other NSUN3 proteins in eukaryotes.

Supplementary Material 2: Table S2: The DEGs of PfNSUN3-Ty1-Ribo strain without GlcN drugs and the 3D7 wild line at the ring, trophozoite, and schizont stages.

Supplementary Material 3: Table S3. The DEGs of PfNSUN3-Ty1-Ribo strain with or without GlcN at the ring, trophozoite, and schizont stages.

Supplementary Material 4: Table S4. Target genes identified by PfNSUN3 enrichment.

Supplementary Material 5: Fig. S1 Sequence alignment of the catalytic domain of the eukaryotic NSUN family. Fig. S2 The PfNSUN3-Ty1-Ribo fusion gene triggered a knockdown effect at the protein level. (A) Western blot of total protein extracts from ring (R), trophozoite (T), and schizont (S) stages from the PfNSUN3-Ty1-Ribo strain with or without culture GlcN. (B–D) Global comparison of expression levels for all genes in the PfNSUN3-Ty1-Ribo line with or without drug at different IDC stages. (E) The transcriptome changes in the *var* gene family of the PfNSUN3-Ty1-Ribo line with or without drug at the ring stage. (F) Histogram displaying the number of up- or downregulated DEGs through the different IDC stages.

## Data Availability

No datasets were generated or analyzed during the current study.

## Abbreviations

IDC: Intraerythrocytic developmental cycle
BSD: Blasticidin S deaminase
GlcN: Glucosamine
RIP-seq: RNA immunoprecipitation and high-throughput sequencing
PAGE: Polyacrylamide gel electrophoresis
GO: Gene Ontology
DEG: Differentially expressed gene

## Declarations

### Permission to reproduce material from other sources

No third-party material requiring formal permission has been used in this study.
