# Supplementary material for: m5C methylation of mitochondrial RNA and non-coding RNA by NSUN3 is associated with variant gene expression and asexual blood-stage development in Plasmodium falciparum
Source: Parasit Vectors. 2025 Mar 27;18:121. doi: 10.1186/s13071-025-06746-7 (PMC11951620; doi:10.1186/s13071-025-06746-7)
Supplement: Supplementary file 5 — Supplementary Material 5: Fig. S1 Sequence alignment of the catalytic domain of the eukaryotic NSUN family. Fig. S2 The PfNSUN3-Ty1-Ribo fusion gene triggered a knockdown effect at the protein level. (A) Western blot of total protein extracts from ring (R), trophozoite (T), and schizont (S) stages from the PfNSUN3-Ty1-Ribo strain with or without culture GlcN. (B–D) Global comparison of expression levels for all genes in the PfNSUN3-Ty1-Ribo line with or without drug at different IDC stages. (E) The transcriptome changes in the var gene family of the PfNSUN3-Ty1-Ribo line with or without drug at the ring stage. (F) Histogram displaying the number of up- or downregulated DEGs through the different IDC stages. [file 13071_2025_6746_MOESM5_ESM.pdf]

Fig. S1

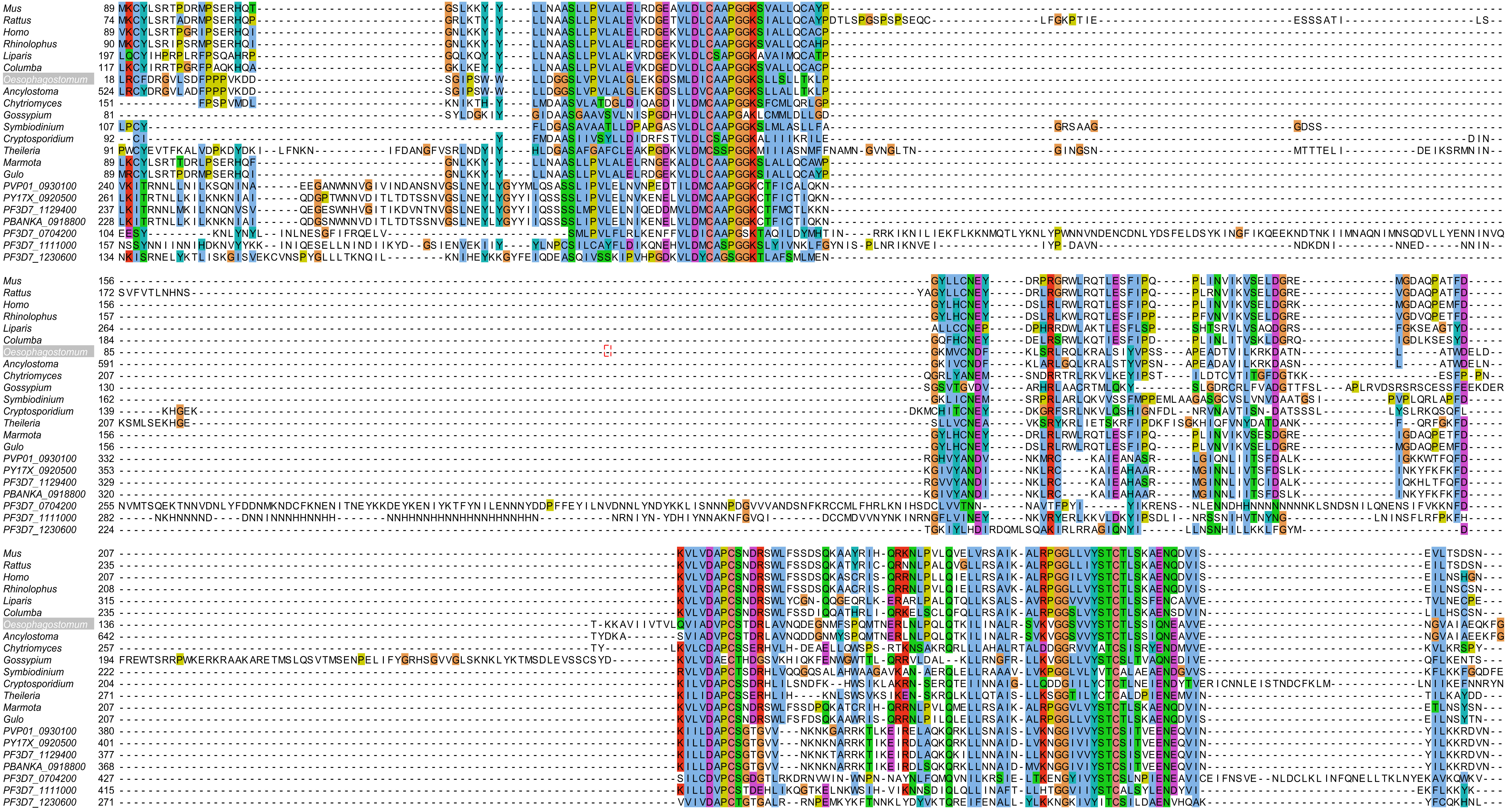
Mus
Rattus
Homo
Rhinolophus
Liparis
Columba
Oesophagostomum
Ancylostoma
Chytriomyces
Gossypium
Symbiodinium
Cryptosporidium
Theileria
Marmota
Gulo
PVP01_0930100
PY17X_0920500
PF3D7_1129400
PBANKA_0918800
PF3D7_0704200
PF3D7_1111000
PF3D7_1230600

Fig. S2

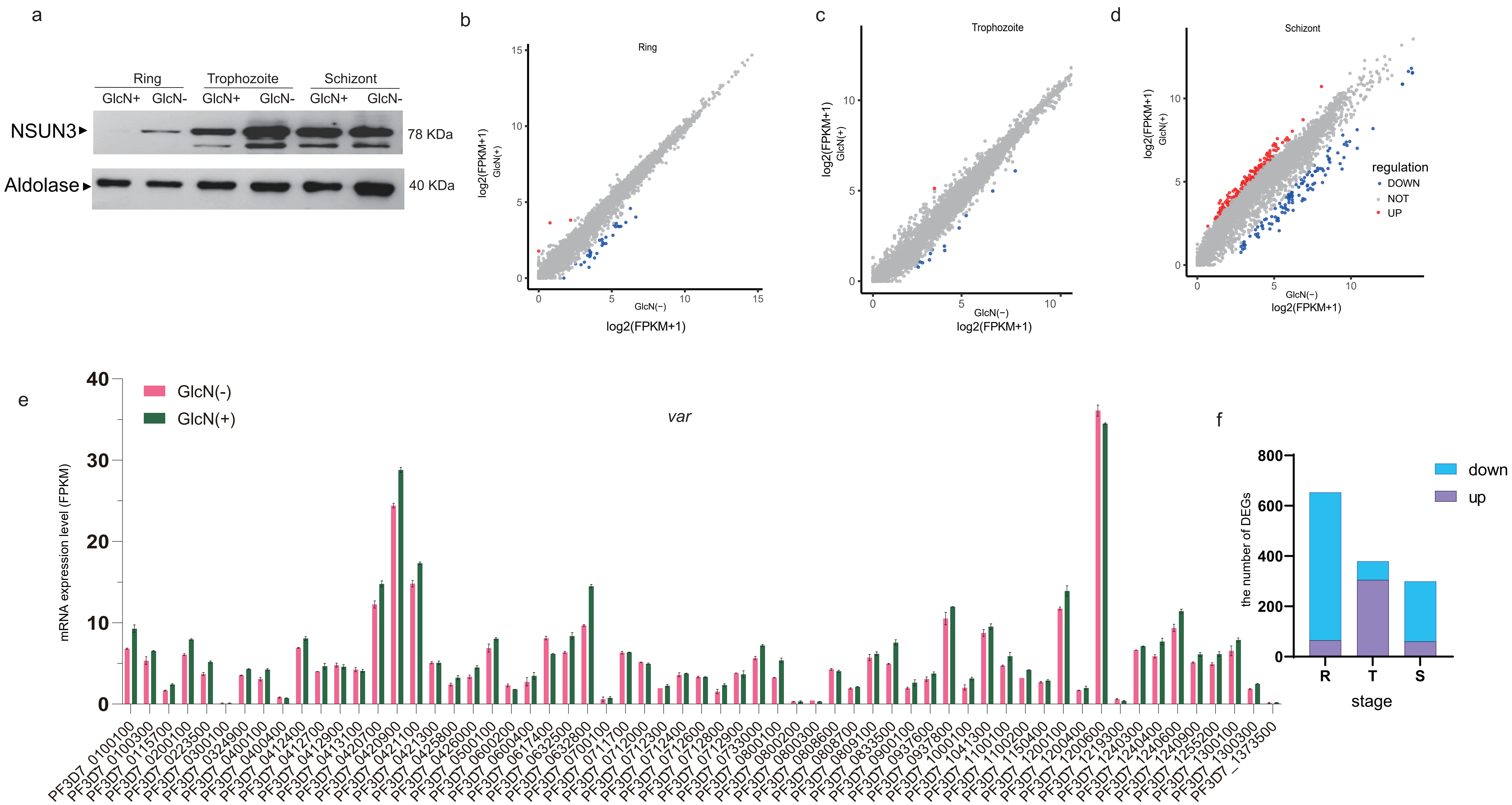

a
Ring
Trophozoite
Schizont
GlcN+ GlcN- GlcN+ GlcN- GlcN+ GlcN-
NSUN3
78 KDa
Aldolase
40 KDa
b
Ring
log2(FPKM+1) GlcN(+)
GlcN(−) log2(FPKM+1)
c
Trophozoite
log2(FPKM+1) GlcN(+)
GlcN(−) log2(FPKM+1)
d
Schizont
log2(FPKM+1) GlcN(+)
GlcN(−) log2(FPKM+1)
regulation
DOWN
NOT
UP
e
GlcN(-)
GlcN(+)
var
mRNA expression level (FPKM)
PF3D7_0100100
PF3D7_0100300
PF3D7_0115700
PF3D7_0200100
PF3D7_0223500
PF3D7_0300100
PF3D7_0324900
PF3D7_0400100
PF3D7_0400400
PF3D7_0412400
PF3D7_0412700
PF3D7_0412900
PF3D7_0413100
PF3D7_0420700
PF3D7_0420900
PF3D7_0421100
PF3D7_0421300
PF3D7_0425800
PF3D7_0426000
PF3D7_0500100
PF3D7_0600200
PF3D7_0600400
PF3D7_0617400
PF3D7_0632500
PF3D7_0632800
PF3D7_0700100
PF3D7_0711700
PF3D7_0712000
PF3D7_0712300
PF3D7_0712400
PF3D7_0712600
PF3D7_0712800
PF3D7_0712900
PF3D7_0733000
PF3D7_0800100
PF3D7_0800200
PF3D7_0800300
PF3D7_0808600
PF3D7_0808700
PF3D7_0809100
PF3D7_0833500
PF3D7_0900100
PF3D7_0937600
PF3D7_0937800
PF3D7_1000100
PF3D7_1041300
PF3D7_1100100
PF3D7_1100200
PF3D7_1150400
PF3D7_1200100
PF3D7_1200400
PF3D7_1200600
PF3D7_1219300
PF3D7_1240300
PF3D7_1240400
PF3D7_1240600
PF3D7_1240900
PF3D7_1255200
PF3D7_1300100
PF3D7_1300300
PF3D7_1373500
f
down
up
the number of DEGs
R
T
S
stage
